# Author Correction: Molecular mechanism of phosphopeptide neoantigen immunogenicity

**DOI:** 10.1038/s41467-023-40274-1

**Published:** 2023-07-27

**Authors:** Yury Patskovsky, Aswin Natarajan, Larysa Patskovska, Samantha Nyovanie, Bishnu Joshi, Benjamin Morin, Christine Brittsan, Olivia Huber, Samuel Gordon, Xavier Michelet, Florian Schmitzberger, Robert B. Stein, Mark A. Findeis, Andy Hurwitz, Marc Van Dijk, Eleni Chantzoura, Alvaro S. Yague, Daniel Pollack Smith, Jennifer S. Buell, Dennis Underwood, Michelle Krogsgaard

**Affiliations:** 1 Department of Pathology, New York University Grossman School of Medicine, New York, NY USA; 2 Laura and Isaac Perlmutter Cancer Center at NYU Langone Health, New York, NY USA; 3 Agenus, Lexington, MA USA

**Keywords:** Tumour immunology, X-ray crystallography, Solution-state NMR, Molecular modelling, MHC

Correction to: *Nature Communications* 10.1038/s41467-023-39425-1, published online 23 June 2023

The original version of this Article omitted from the author list the 16^th^, 17^th^ and 18^th^ authors Eleni Chantzoura, Alvaro S. Yague, Daniel Pollack Smith, who are from ‘Agenus, Lexington, MA, USA’. Consequently, the following was added to the Competing Interest section: ‘D.U., B.M., X.M., B.J., C.B., O.H., S.G., E.C, A.S.Y., F.S., R.B.S., M.A.F., A.H., D.P., M.v.D., J.S.B. have ownership of equity securities and/or are currently or previously employed by Agenus.’ Additionally, the following was added to the Author Contributions: ‘T-SPRINT data analysis: A.S.Y. (investigation)’ as well as ‘X.M., D.P. and E.C. conceived, designed and performed the cell-based experiments.’. This has been corrected in both the PDF and HTML versions of the Article.

