# Supplementary material for: Molecular mechanism of phosphopeptide neoantigen immunogenicity
Source: Nat Commun. 2023 Jun 23;14:3763. doi: 10.1038/s41467-023-39425-1 (PMC10290117; doi:10.1038/s41467-023-39425-1)
Supplement: Supplementary file 1 — Supplementary Information [file 41467_2023_39425_MOESM1_ESM.pdf]

X-ray diffraction data and refinement statistics

* - data for high resolution shells are in parentheses

Supplementary Table 1

**Property/PDB ID**	**7RZD**	**7S8F**	**7RZJ**	**7S8A**	**7S8E**	**7S79**	**7S7D**	**7S7E**	**7S7F**	**7S8I**	**7S8J**
Type of protein assembly	pMHC-I	pMHC-I with bound glycerol	pMHC-I #1	pMHC-I #2	pMHC-I with bound glycerol	pMHC-I	pMHC-I	pMHC-I	pMHC-I	TCR_1	TCR_2
Peptide Ligand	MLL	MLL	pMLL	pMLL	pMLL	phosphono-MLL	sulfo-MLL	DOT1L	pDOT1L	none	none
X-Ray source	NSLS-II 17-ID1	APS 19BM	NSLS-II 17-ID1	NSLS-II 17-ID1	APS 19BM	NSLS-II 17-ID1	NSLS-II 17-ID1	NSLS-II 17-ID1	NSLS-II 17-ID1	APS 19BM	APS 19BM
Space group	$P 4_1 2_1 2$	$P 4_1 2_1 2$	$P 4_1 2_1 2$	$I2_13$	$P 4_1 2_1 2$	$P 4_1 2_1 2$	$P 4_1 2_1 2$	$P 4_1 2_1 2$	$P 4_1 2_1 2$	$P 1 2_1 1$	$P 2_1 2_1 2_1$
Cell constants, a b c (Å)	65.90 65.90 238.90	65.57 65.57 238.23	65.72 65.72 238.48	147.81 147.81 147.81	65.55 65.55 238.14	65.86 65.86 238.86	65.33 65.33 237.61	65.30 65.30 238.53	65.30 65.30 238.53	39.68 77.23 67.68	60.55 73.33 110.03
Angles, α β γ (^{0})	α=β=γ=90	α=β=γ=90	α=β=γ=90	α=β=γ=90	α=β=γ=90	α=β=γ=90	α=β=γ=90	α=β=γ=90	α=β=γ=90	90.00 104.38 90.00	α=β=γ=90
Resolution range (Å)	29.47–1.82	46.37-1.80	29.17-1.80	29.01-2.10	45.54-1.60	29.25-1.53	29.72-1.56	29.20-2.04	29.43-1.88	33.71-1.66	29.21-1.92
Completeness, %*	99.96(98.5)	99.8(100)	99.9(99.8)	99.9(95.0)	100(100)	99.8(97.7)	98.2(81.7)	99.9(98.6)	99.8(97.2)	98.4(85.1)	99.6(95.3)
Redundancy*	14.6(14.7)	6.8(6.6)	10.8(10.3)	11.5(11)	6.8(6.6)	8.7(8.5)	8.0(4.3)	10.9(10.2)	13.2(13.4)	3.2(2.4)	3.2(5.5)
R_{merge}	0.07	0.12	0.12	0.17	0.09	0.05	0.07	0.14	0.11	0.05	0.09
I/σ(I)*	22.8(3.8)	12.5(2.3)	11.7(2.4)	10.1(2.9)	12.3(1.9)	22.4(2.7)	15.1(2.0)	11.6(2.8)	14.7(3.5)	14.1(2.1)	5.6(2.3)
R, R_{free}	0.179, 0.236	0.163,0.206	0.166,0.217	0.160,0.206	0.168,0.195	0.170,0.196	0.166,0.195	0.172,0.234	0.168,0.217	0.167,0.216	0.168,0.213
R_{free} reflections test set (%)	1474(3.0)	1569(3.2)	1581(3.2)	939(3.0)	2118 (3.0)	2491(3.1)	2213(3.0)	1007(3.0)	1386(3.2)	1421(3.1)	1156(3.1)
F_o,F_c correlation	0.97	0.96	0.96	0.96	0.97	0.97	0.97	0.96	0.96	0.97	0.96
Total number of atoms	3833	3805	3767	3580	3842	3902	3827	3660	3795	4065	3930
Average B factor, all atoms (Å^2)	36	30	31	33	27	29	28	38	33	33	38
Ramachandran plot:											
Most favored, %	96.4	96.47	97.47	96.55	96.9	97.38	96.83	96.57	97.69	95.34	94.82
Allowed, %	3.6	3.24	2.53	2.92	2.82	2.62	3.17	3.43	2.31	4.41	5.18
Disallowed, %(Number of residues)	0	0.29(1)	0	0.53(2)	0.28(1)	0	0	0	0	0.25(1)	0
RMS deviations:											
Bonds, Å	0.007	0.008	0.008	0.008	0.008	0.01	0.008	0.007	0.007	0.01	0.007
Angles, ^{0}	1.454	1.436	1.429	1.471	1.488	1.571	1.431	1.482	1.443	1.684	1.439

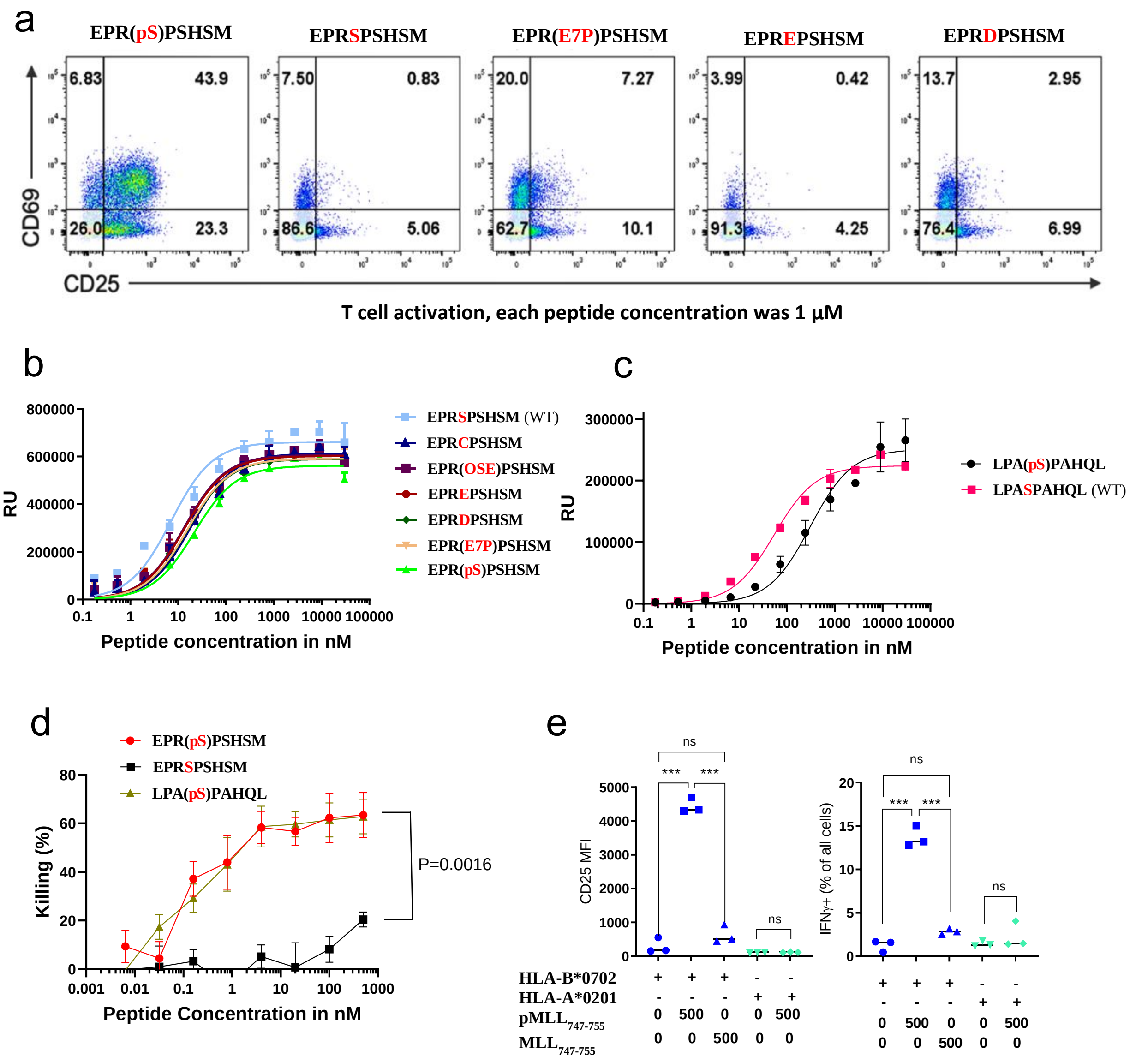

Supplementary Figure 1. Phosphoserine P_4 has a key role in TCR27-dependent T cell activation. Residues at P_4 are colored . All the measurements in **b-e** were made in triplicates and expressed as mean values or mean +/- SEM. Non-linear fit in **b-c** was performed in GraphPad.

a T cell activation assay. T2 cells expressing HLA-B*0702 were pulsed with various concentrations of $pMLL_{747-755}$ peptide or analogues, the AA sequences of which varied at P_4, and co-cultured with TCR27-transduced T cells or with control non-transduced T cells overnight. Activated T cells were identified by surface expression of CD25 and CD69 using flow cytometry.

b Binding between HLA-B*0702 and $pMLL_{747-755}$ or its mutants with substituted residue P_4 was measured using an Alpha assay.

c Binding between HLA-B*0702 and $pDOT1L_{998-1006}$ or $DOT1L_{998-1006}$ was measured using an Alpha assay.

d Concentration- and phosphoserine-dependent killing of HLA-B*0702+ T2 cells pulsed with the indicated peptides by the TCR27+ T cells was determined by LIVE/DEAD viability staining kit. The shown in figure *P* value between the $pMLL_{747-755}$ and $MLL_{747-755}$ datasets was calculated using a two-sided Mann–Whitney U test.

e HLA-epitope matching: T2 cells expressing either HLA-B*0702 or HLA-A*0201 (ATCC CRL1991) were pulsed with 0.5µM of $pMLL_{747-755}$ or $MLL_{747-755}$, respectively, and co-cultured with TCR27-transduced or control (non-transduced) T cells. Activation markers CD25 and IFN gamma were detected by flow cytometry. The p value was calculated using a two-sited t-test, *** p<0.001, ns – not significant.

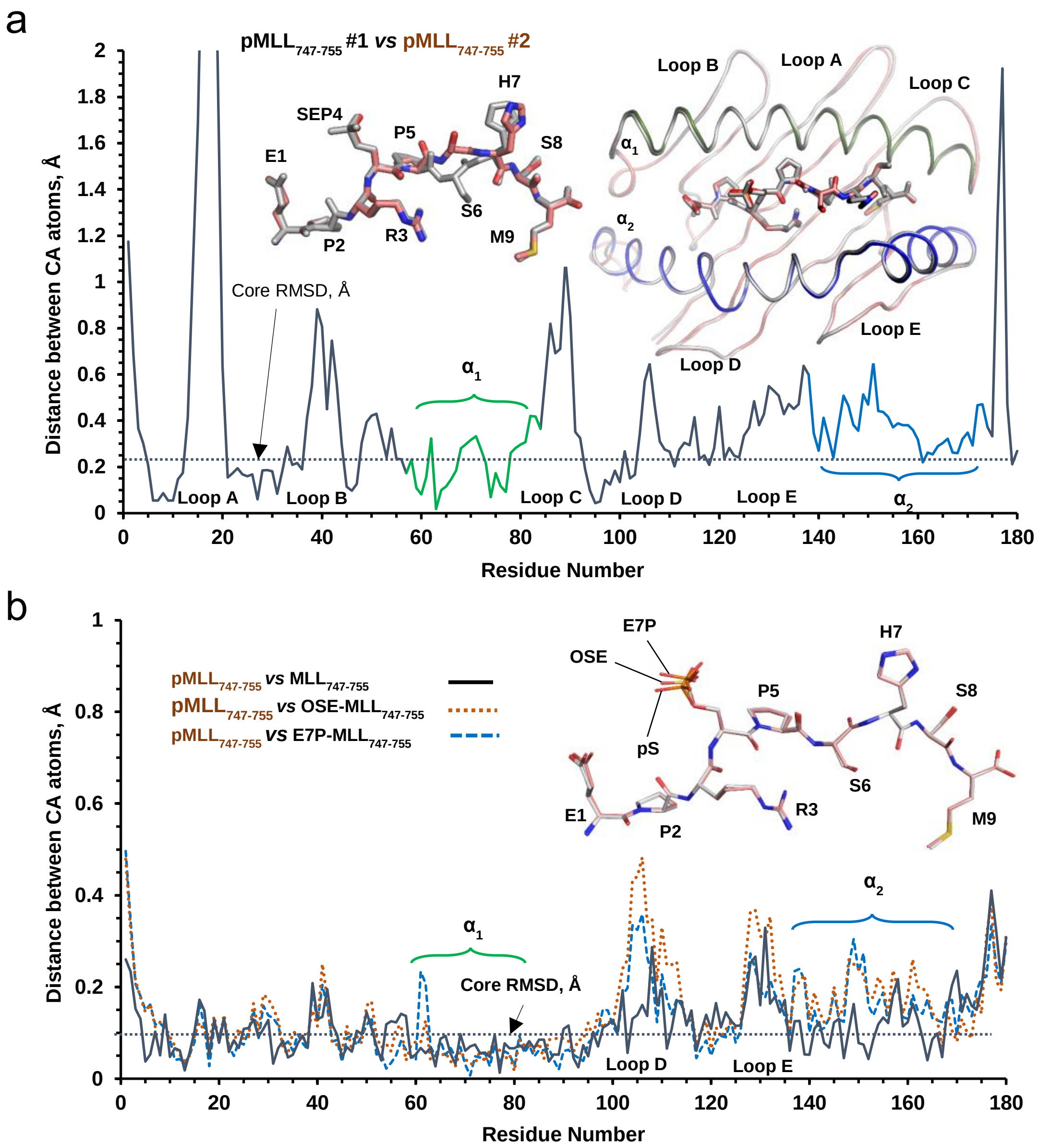

Supplementary Figure 2. Isomorphous pMHC crystal structures with MLL747-75, pMLL747-755 and phosphomimetics share high similarity. The pMHC structures were aligned using the atomic coordinates of the α_1 helices (AA residues 57-85, Cα (CA) carbons only). The horizontal dotted line in each figure indicates a core average RMSD value between the truncated structures. The protein loops were arbitrarily assigned. Peptides are presented as sticks. The two helical regions are designated as α_1 and α_2.

a CA-distance plot between the two non-isomorphous pMLL747-755/HLA-B*0702 structures (#1 and #2). Insets - superimposed peptides as stick models, structure #1 is gray. The cartoon representation shows an overall peptide-binding domain view including bound peptides.

b CA-distance plots between the HLA-B*0702 structures in complex with pMLL747-755, MLL747-755, E7P-MLL747-755, or OSE-MLL747-755. The average core RMSD between these structures was 0.1 Å (dotted line). A slightly elevated displacement was associated with protein loops D and E. Inset - superimposed peptides as stick models.

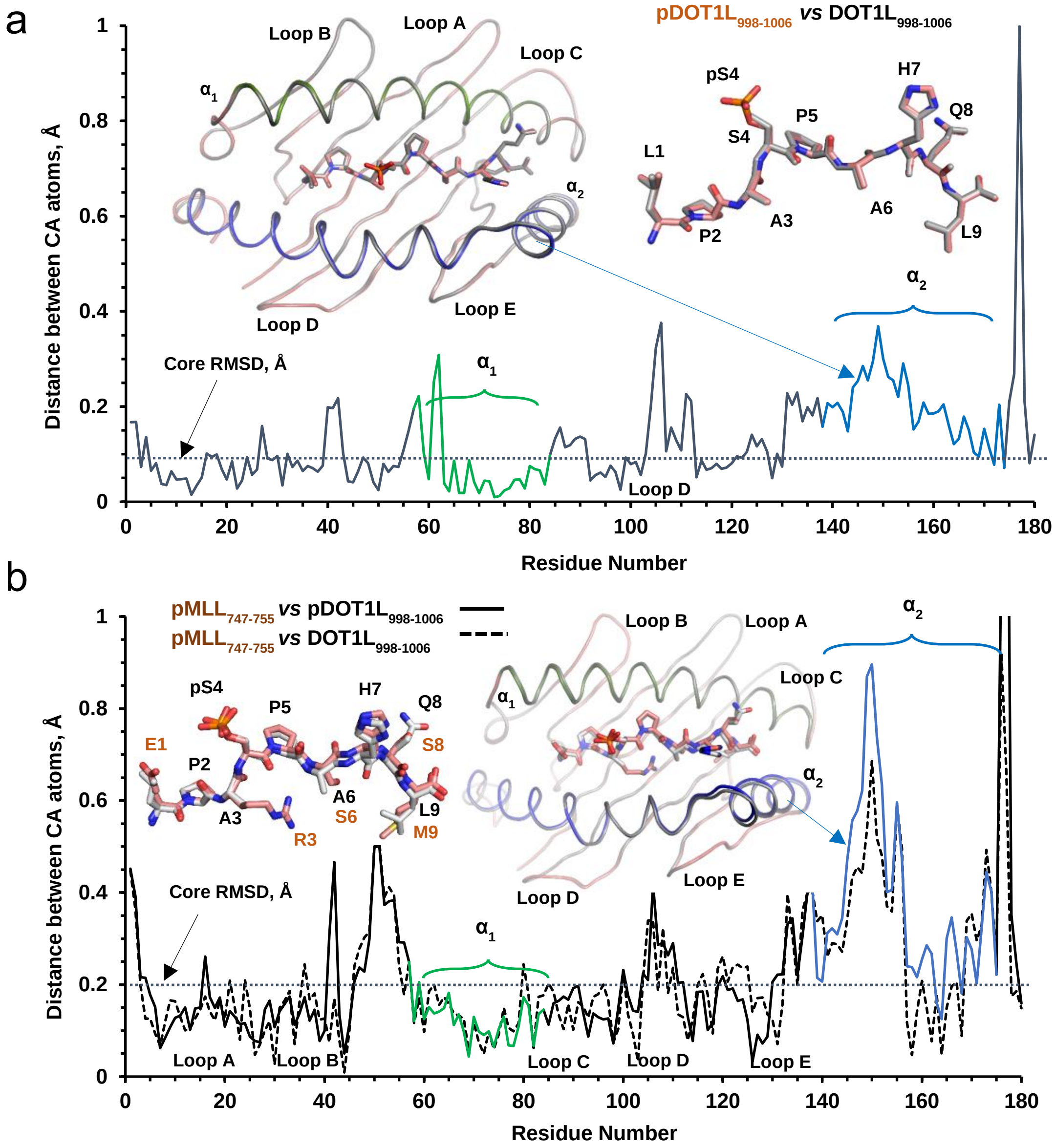

Supplementary Figure 3. pMHC crystal structures with distinct phosphopeptides display local conformational differences. Designations are the same as in Supplementary Figure 2.

a CA-distance plot between the HLA-B*0702 structures in complex with $pDOT1L_{998-1006}$ or $DOT1L_{998-1006}$. Inset – overall domain structure (cartoon), peptides are presented as sticks.

b CA-distance plots between the isomorphous structures for HLA-B*0702 in complex with $pMLL_{747-755}$, $DOT1L_{998-1006}$, or $DOT1L_{998-1006}$. Inset - the cartoon view of superimposed structures with $pMLL_{747-755}$ or $pDOT1L_{998-1006}$. Inset – superposition of $pMLL_{747-755}$ and $pDOT1L_{998-1006}$, stick models.

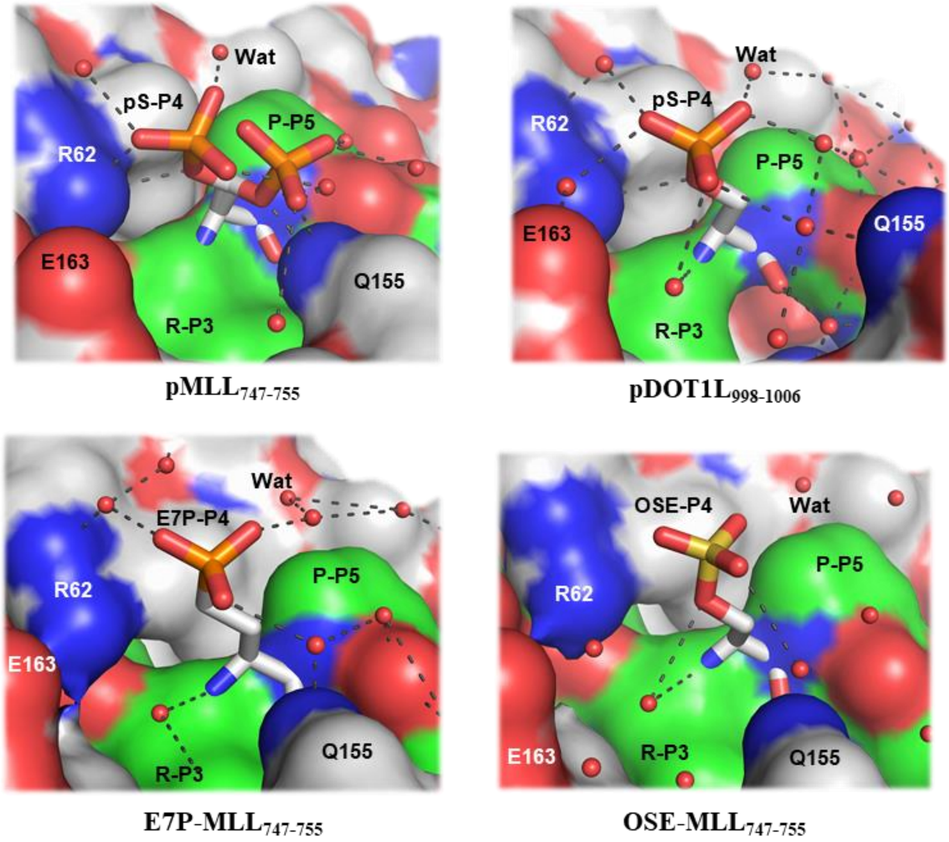

Supplementary Figure 4. The polar side chain of residue P_4 adopts a non-anchor orientation and remains solvent-exposed in every pMHC structure. Surface representation. The HLA residues are colored according to the atomic properties (N - blue, O - red, C - light grey). The epitope is shown as a surface model, its carbon atoms are colored in green except for P_4, which is displayed as a stick model. Water molecules are the non-bonded spheres, H-bonds are the dotted lines.

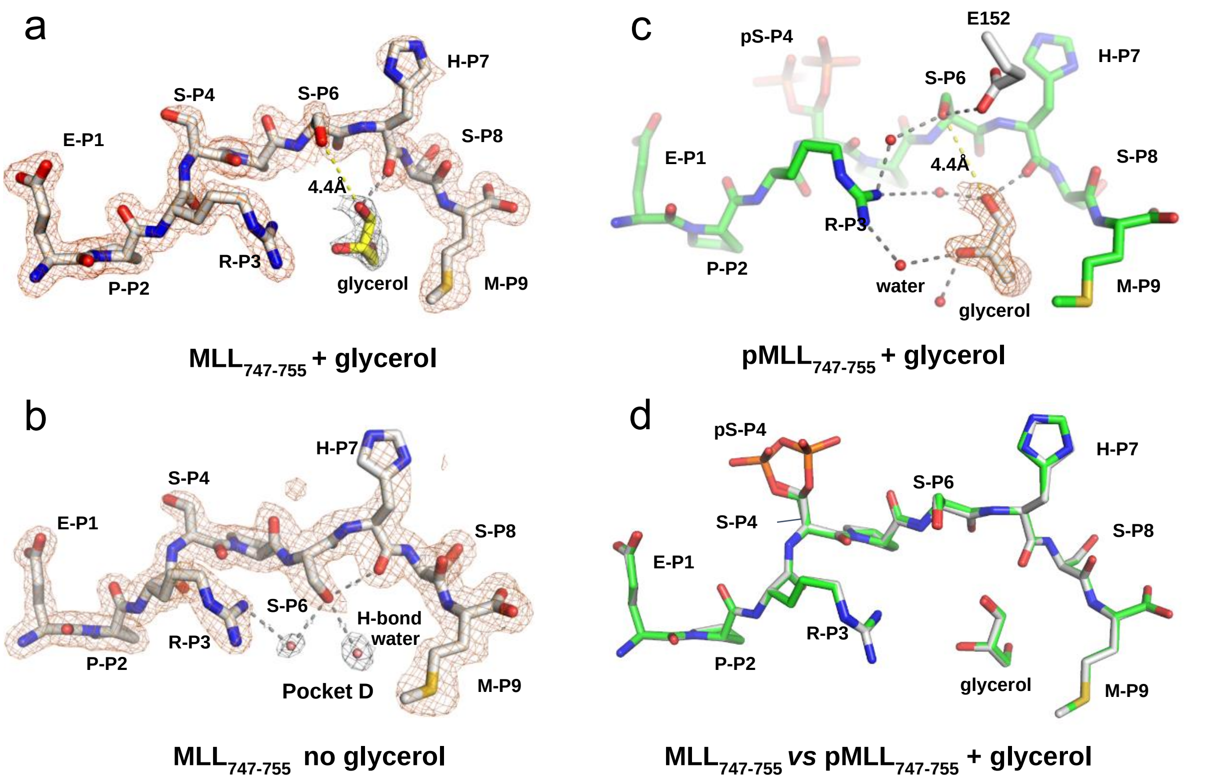

Supplementary Figure 5. Glycerol affects peptide conformation and expels water from the pMHC crystal structure. For all plots, the peptide residues are shown as sticks, the *sigmaA*-weighted 2Fo-Fc electron density maps (σ=1.0, cutoff radius = 1.5 Å) are drawn as mesh around selected residues. The hydrogen bonds are dotted lines, other distances are depicted by yellow dashes. Waters are presented as non-bonded spheres.

a HLA-B*0702 structure in complex with $MLL_{747-755}$ and bound glycerol; carbons of glycerol are yellow-colored.

b HLA-B*0702 structure in complex with $MLL_{747-755}$ without glycerol but with bound water.

c HLA-B*0702 structure in complex with $pMLL_{747-755}$ and bound glycerol.

d Superposition of the HLA-B*0702 structures in complex with $pMLL_{747-755}$ or $MLL_{747-755}$ (both with bound glycerol) shows very high similarity between the conformations of the two peptides.

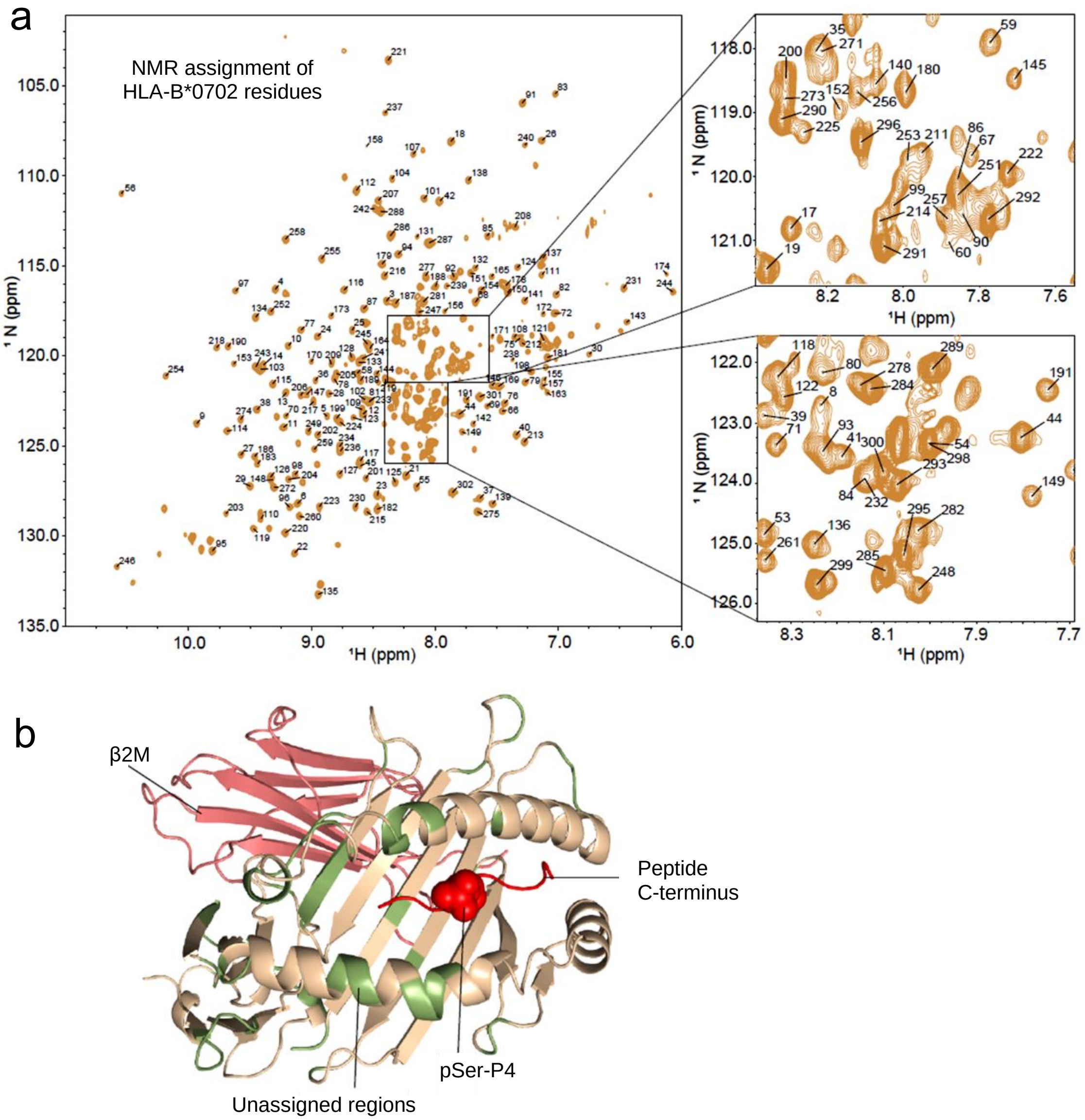

Supplementary Figure 6. NMR assignments of amino acid residues of HLA-B*0702 complexed with pMLL$_{747\text{-}755}$.

a ^{15}N-TROSY spectra of pMLL$_{747\text{-}755}$/HLA-B*0702 with assignments depicted on the spectrum with residue numbers. Boxes – zoomed-in view of the central crowded regions of the ^{15}N-TROSY spectra of the pMLL$_{747\text{-}755}$/HLA-B*0702 complex with assignments depicted.

b Cartoon representation of the pMLL$_{747\text{-}755}$/HLA-B*0702 crystal structure with NMR-assigned regions indicated in cream. The peptide is red with pSer-P$_4$ atoms represented as spheres and unassigned regions are green. A total of 86% of all assignable (excluding proline residues) backbone N and HN HLA-B*0702 atoms were assigned and 84% of all residues of the alpha helical regions that surround the bound peptide were assigned. Some residues could not be assigned due to peak overlaps.

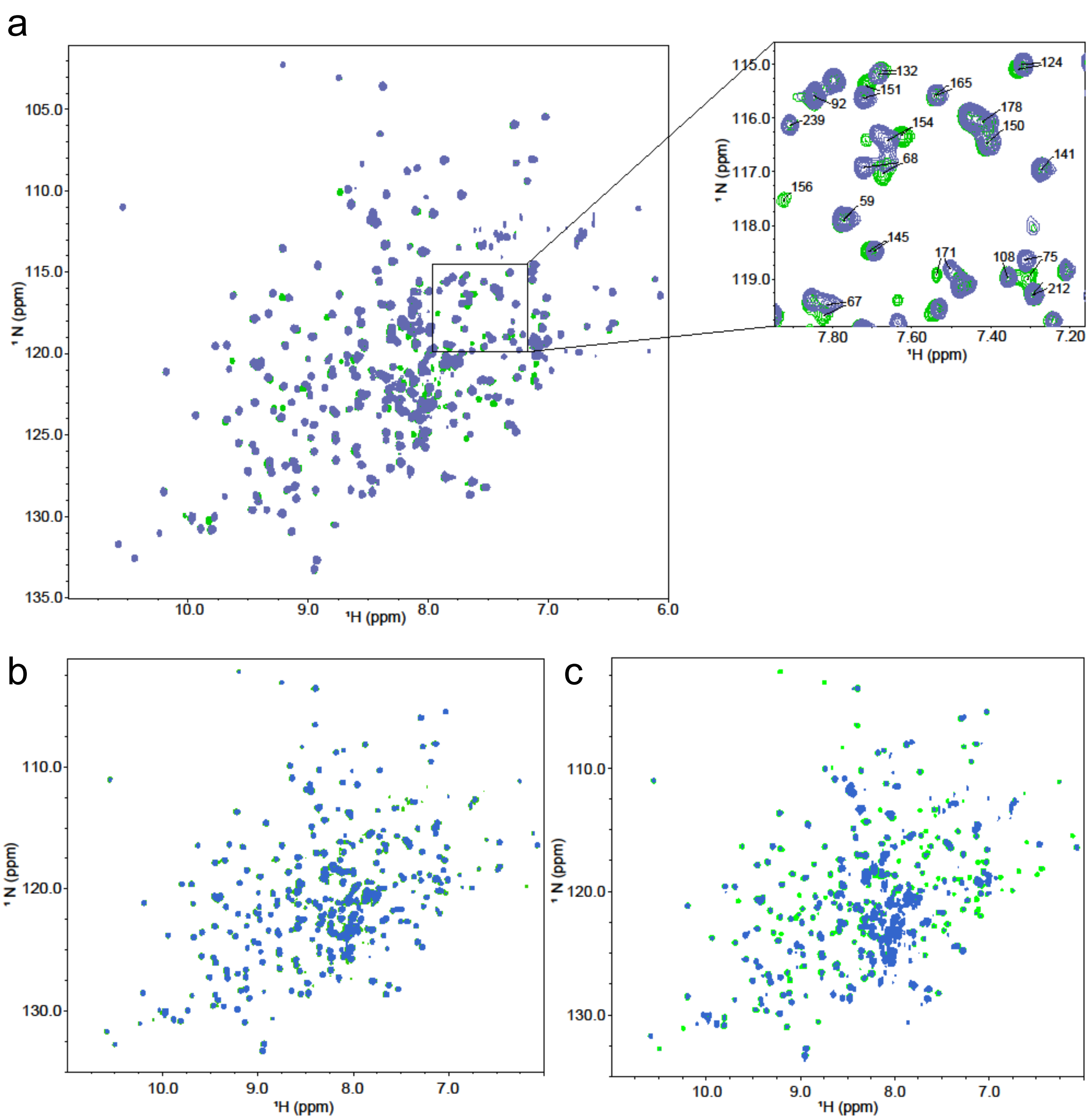

Supplementary Figure 7. NMR spectra of pMLL$_{747-755}$/HLA-B*0702 or MLL$_{747-755}$/HLA-B*0702 alone or in the presence of TCR27.

a Overlay of the ^{15}N-TROSY spectra for pMLL$_{747-755}$/HLA-B*0702 (green) and MLL$_{747-755}$ /HLA-B*0702 (dark blue) complexes. Box – zoomed-in view of the overlay highlighting chemical shift perturbations of the individual residues located at or close to the peptide-HLA interface.

b Overlay of the ^{15}N-TROSY spectra for MLL$_{747-755}$/HLA-B*0702 (green) and for TCR27: MLL$_{747-755}$/HLA-B*0702 mixture (1:1 molar ratio, blue).

c Overlay of the ^{15}N-TROSY spectra for pMLL$_{747-755}$/HLA-B*0702 (green) and for TCR27: pMLL$_{747-755}$/HLA-B*0702 mixture (1:1 molar ratio, blue). Significant drop in peak intensities indicates complexation between TCR and pMHC.

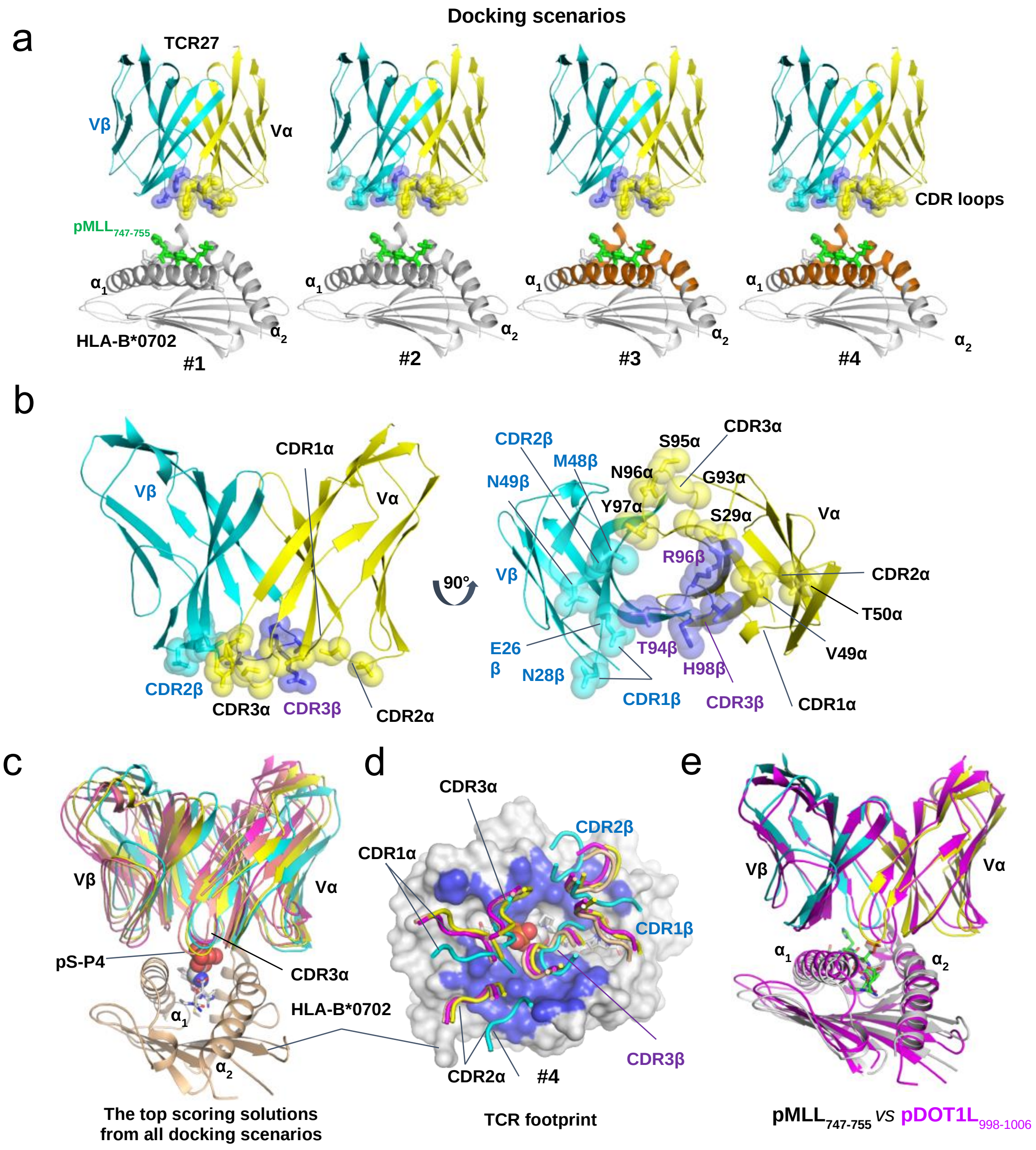

Supplementary Figure 8. **Haddock docking strategy and TCR-pMHC docking.** The TCR27-pMHC truncated model included the variable region of TCR27 (cartoon), the peptide binding domain of HLA-B*0702 (AA residues 1-180, cartoon) and pMLL$_{747\text{-}755}$ peptide (stick model).

a Different guided docking scenarios (1-4). The selected residues in HLA and pMLL$_{747\text{-}755}$ are colored, and those not selected are grey. The residues selected in TCR27 are shown as sticks and semi-transparent spheres.

b Residues in CDR loops (TCR_1) that were selected for guided docking (the two figures are related by about 90° rotation) are drawn as sticks and semi-transparent spheres and colored similarly to the corresponding protein chains.

c Superposition of the top Haddock solutions from docking scenarios 1-4 (with pMLL$_{747\text{-}755}$ peptide). The coordinates of the pMHC atoms were aligned to demonstrate the relative orientation of TCR27 with respect to the pMHC in each ternary complex. The maximum RMSD value between these models was 3.1Å and the minimum value was 0.9Å.

d Composite TCR footprint (combined from the 4 top solutions) over the pMHC (surface model, gray) shows the HLA-B*0702 areas (shaded dark blue) located at the TCR-pMHC interface (inter-atomic distance cutoff 4Å). The individual CDR loops (cartoons) are labeled, pMLL$_{747\text{-}755}$ is a stick model, the phosphate group is shown as spheres, and the docking scenario #4 is designated (light blue).

e Comparison of the top docking solutions with pMLL$_{747\text{-}755}$ or pDOT1L$_{998\text{-}1006}$ epitopes. Alignment of the top solutions from scenario #4 was performed as in (**b**); the RMSD value between all the aligned atoms was 1.9Å.

a

Interacting residues selected for docking

Location	Res #, MHC	Location	Res #, TCR
Peptide	pSer-P4	CDR1α	Ser29
Peptide	Pro-P4	CDR2α	Val49
Peptide	Ser-P6	CDR2α	Thr50
Peptide	His-P7	CDR3α	Tyr91
HLA-B*0702	Asp61	CDR3α	Gly92
HLA-B*0702	Arg62	CDR3α	Gly93
HLA-B*0702	Ile66	CDR3α	Gly94
HLA-B*0702	Lys68	CDR3α	Ser95
HLA-B*0702	Ala69	CDR3α	Asn96
HLA-B*0702	Gln70	CDR3α	Tyr97
HLA-B*0702	Thr73	CDR1β	Asn26
HLA-B*0702	Glu76	CDR1β	Glu28
HLA-B*0702	Trp147	CDR2β	Met48
HLA-B*0702	Ala149	CDR2β	Asn49
HLA-B*0702	Arg156	CDR3β	Thr94
HLA-B*0702	Ala158	CDR3β	Gly95
HLA-B*0702	Glu163	CDR3β	Arg96
		CDR3β	Val97
		CDR3β	His98

c

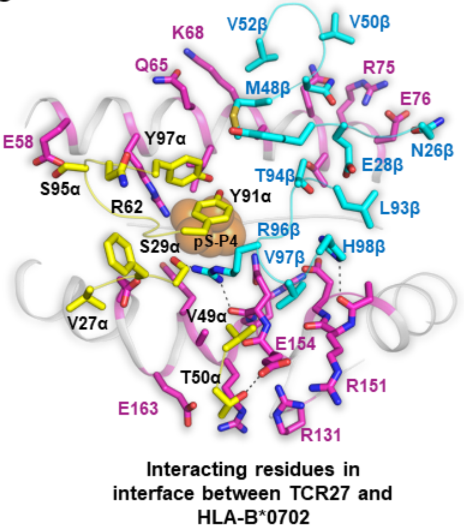

b

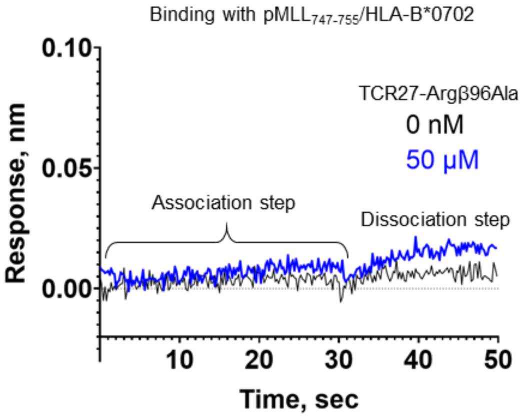

d

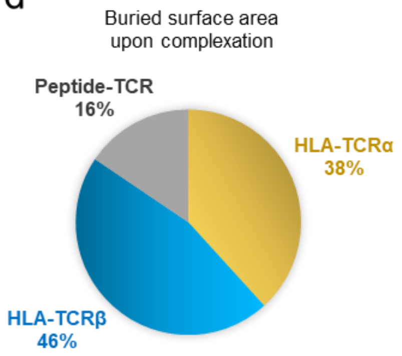

Supplementary Figure 9. The role of HLA-B*0702 in complexation between pMHC and TCR27.

a List and location of interacting residues used in Haddock docking. Residues were selected based on the various identification methods, described in the text.

b BLI sensogram shows no interaction between the TCR27 Arg96Ala mutant (solute) and the $pMLL_{747\text{-}755}$/HLA-B*0702 binary complex (ligand).

c Interface between TCR27 and pMHC (the interatomic distance cutoff 4Å), in which interacting residues are shown as sticks with their carbon atoms colored according to the protein chains. Peptide $pMLL_{747\text{-}755}$ is shown as a gray cartoon with the phosphate group displayed as a space-filled model. Other TCR-interacting residues in $pMLL_{747\text{-}755}$ were omitted for clarity and to outline the role of HLA residues in TCR-pMHC complexation.

d Diagram showing the buried surface area upon complexation between TCR27 and pMHC ($pMLL_{747\text{-}755}$/HLA-B*0702) with contributions from the different protein components.

Output data from the Haddock scenario #4

**Property**	**Cluster 1**	**Cluster 2**
HADDOCK score	**-143.8 +/- 2.3**	**-105.7 +/- 4.3**
Cluster size	**154**	**15**
RMSD from the overall lowest-energy structure	**0.6 +/- 0.4**	**17.1 +/- 0.4**
Van der Waals energy	**-58.8 +/- 4.1**	**-52.7 +/- 7.0**
Electrostatic energy	**-407.4 +/- 10.1**	**-259.4 +/- 26.8**
Desolvation energy	**-4.7 +/- 1.5**	**-5.4 +/- 1.6**
Restraints violation energy	**11.6 +/- 3.2**	**42.9 +/- 22.3**
Buried Surface Area	**1868.0 +/- 66.0**	**1776.2 +/- 28.9**
Z-Score	**-1.4**	**0.6**

Supplementary Figure 10. Output data from a Haddock run, scenario #4, with docking statistics for the top two clusters of docking solutions. Data are presented as mean values +/- SEM.

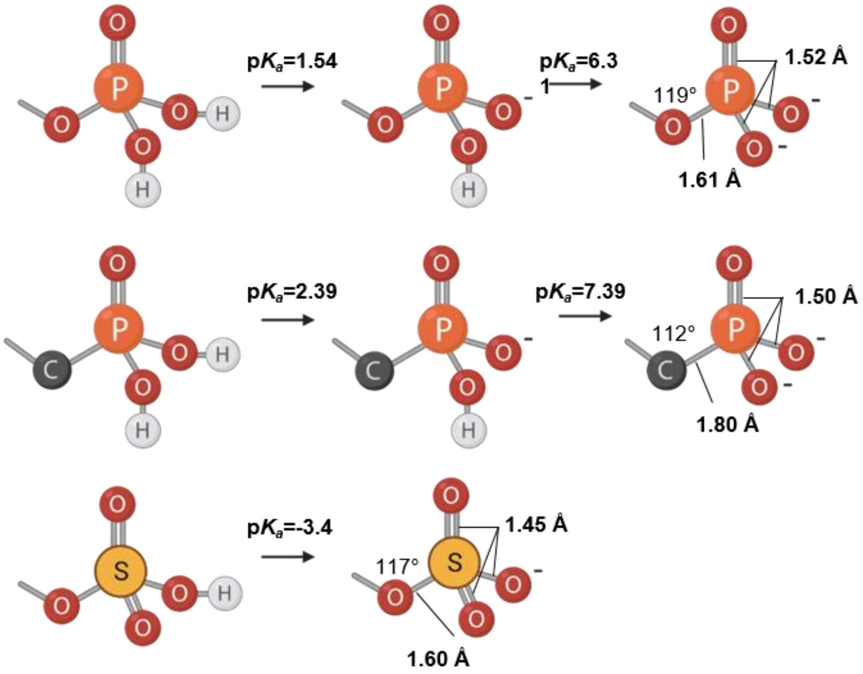

Supplementary Figure 11. The similarities and differences between phosphate and phosphomimetics. The electronic structures, charges and pK_a values for alkyl-derivatives of phosphate, phosphonate and sulfate. According to Elliott et al., 2012.

Reference: Elliott TS, Slowey A, Ye Y, Conway SJ. The use of phosphate bioisosteres in medicinal chemistry and chemical biology. MedChemComm **3**, 735-751 (2012).

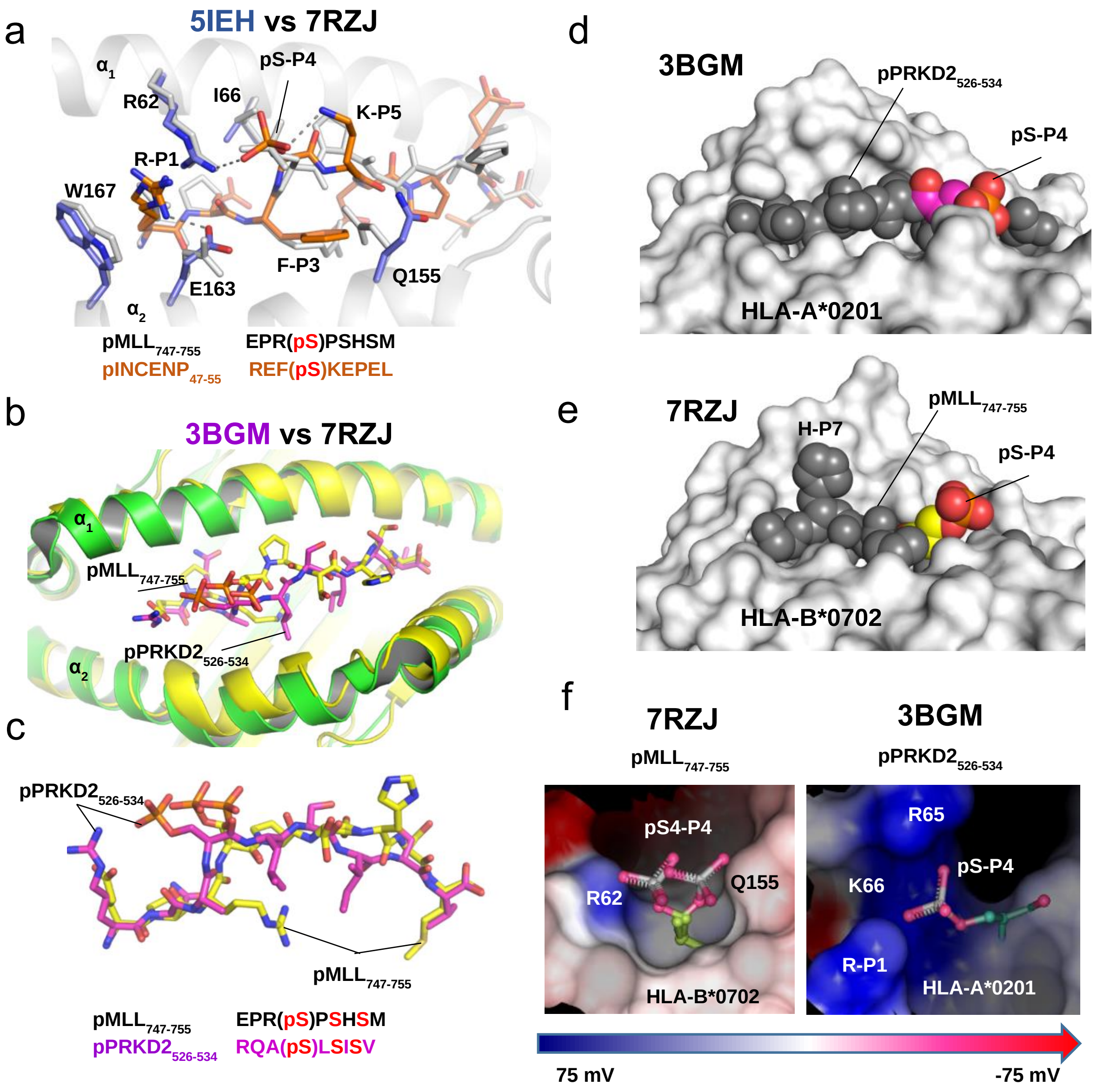

Supplementary Figure 12. PMHC conformation is dependent on HLA type and amino acid sequence of the bound phosphopeptide. Hydrogen atoms were omitted for clarity.

a Superposition of HLA-B*4001 in complex with phosphoepitope pINCENP$_{47-55}$ (Alpizar A. et al., 2017; PDB 5IEH, carbon atoms are colored) and HLA-B*0702 in complex with pMLL$_{747-755}$ (PDB 7RZJ, all atoms are grey) shows a similar orientation for pSer-P$_4$.

b Superposition of the structures for HLA-A*0201 in complex with phosphoepitope pPRKD2$_{526-534}$ (PDB 3BGM, Mohammed F. et al., 2008; carbon atoms are in magenta and green) and HLA-B*0702 in complex with pMLL$_{747-755}$ (PDB 7RZJ, carbon atoms are yellow) shows differences between the conformations of bound epitopes (residues P$_4$-P$_8$), including that of pSer-P$_4$.

c Zoomed-in view of the bound phosphopeptides from (**b**). Identical AA residues are red colored.

d and **e** Different orientations of pSer-P4 residues in the two phosphopeptides lead to distinct peptide conformations and likely reduced solvent exposure of the phosphate group in the 3BGM structure, as compared to 7RZJ. Each HLA structure is displayed as surface. Peptide atoms are shown as spheres, the pSer-P$_4$ residue is colored.

f HLA surface in the structures 7RZJ and 3BGM is colored according to the charge. The residues maintaining the H-bond with the phosphate group are designated.

References: Alpizar A, et al. A Molecular Basis for the Presentation of Phosphorylated Peptides by HLA-B Antigens. Mol Cell Proteomics **16**, 181-193 (2017). Mohammed F, et al. Phosphorylation-dependent interaction between antigenic peptides and MHC class I: a molecular basis for the presentation of transformed self. Nat Immunol **9**, 1236-1243 (2008).

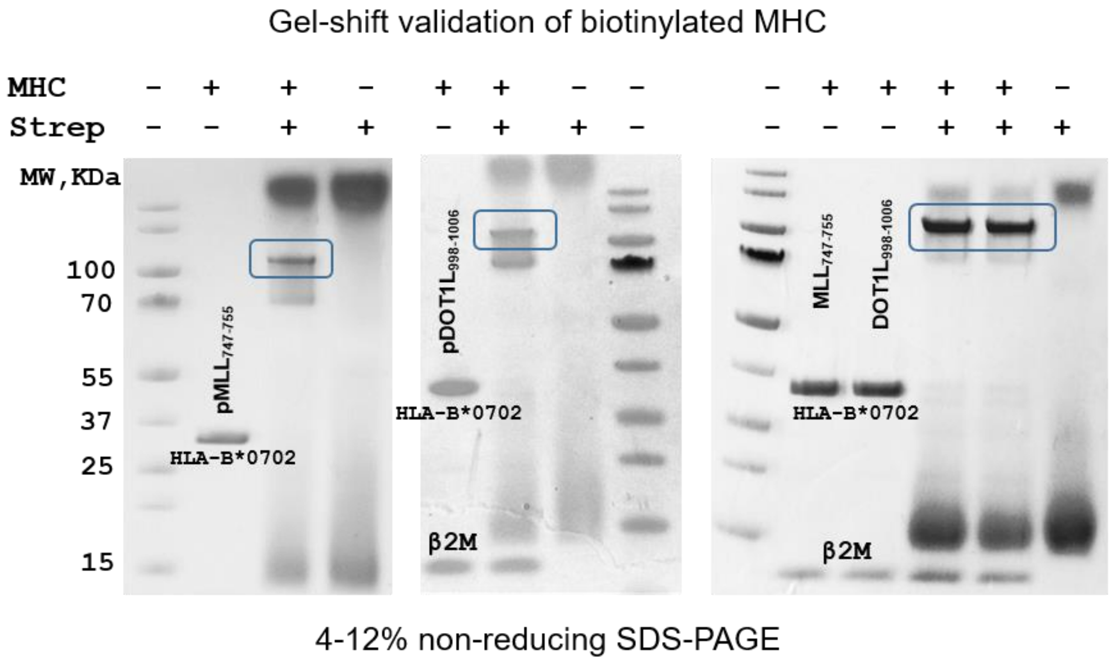

Supplementary Figure 13. An example of a gel-shift assay confirming protein biotinylation. For testing, each pMHC preparation with biotinylated HLA-B*0702 was mixed with streptavidin in a molar ratio of around 1:2, incubated for 20 min at room temperature and analyzed using non-reducing SDS-PAGE. Controls were not mixed. The position of the HLA-Streptavidin complex is outlined. Data reproducibility and protein stability in each case were evaluated as presented here 24 hours and 7 days post-biotinylation (storage at +4^{0}C). No visual changes in proteins stability were detected.

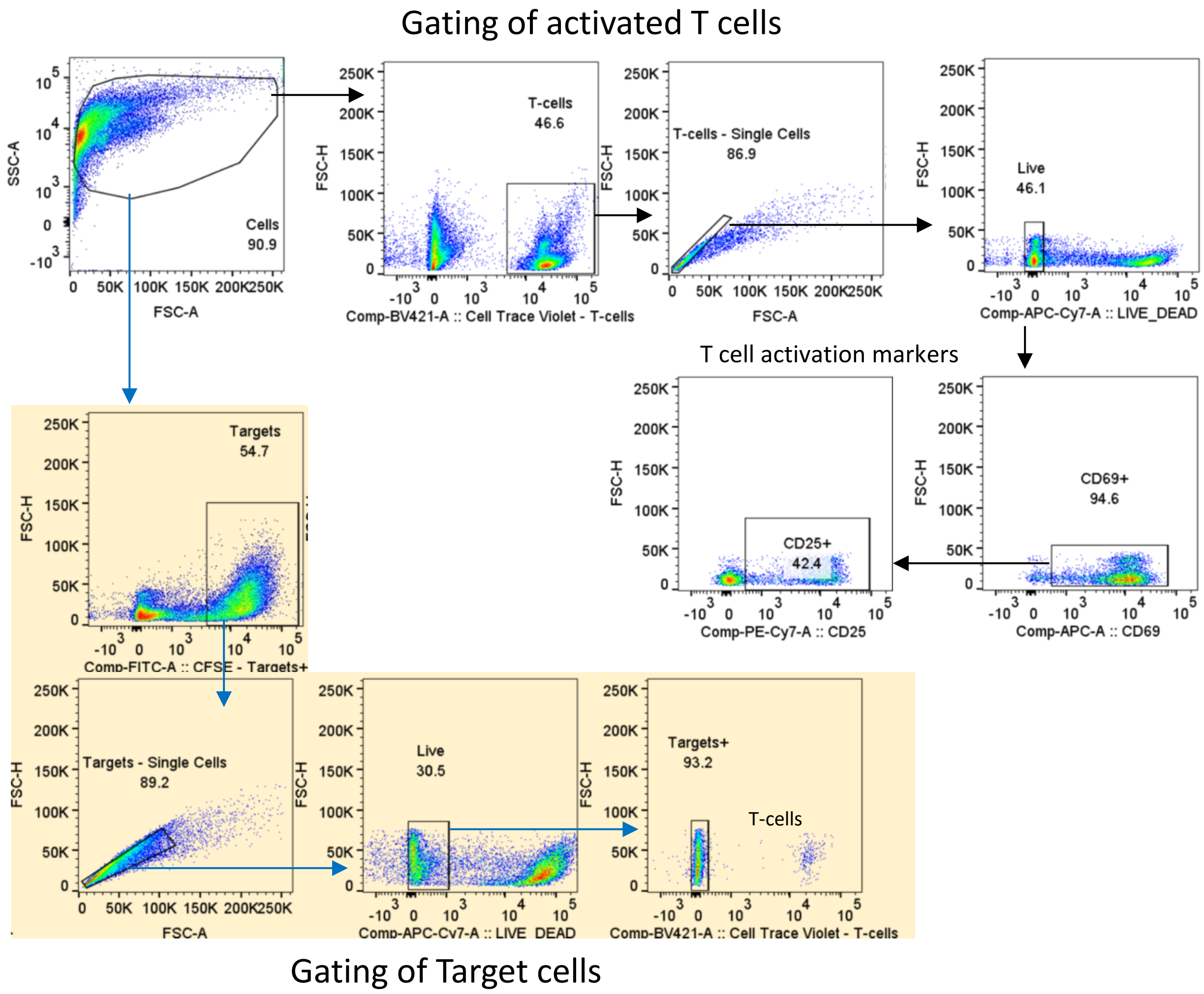

Supplementary Figure 14. Flow cytometry gating strategy. Cells were gated on single Lymphocytes (or single cells) based on size; the T cells and Target cells were separated using the proliferation markers, Cell Trace Violet and CFSE, respectively. Single cells were gated using FSC-A and FSC-H, then dead cells were gated using a Live/Dead marker. Then for the T cells, a co-expression of activation-induced markers CD25 and CD69 was identified.
