# Supplementary material for: Molecular mechanism of phosphopeptide neoantigen immunogenicity
Source: Nat Commun. 2023 Jun 23;14:3763. doi: 10.1038/s41467-023-39425-1 (PMC10290117; doi:10.1038/s41467-023-39425-1)
Supplement: Supplementary file 3 — Description of Additional Supplementary Files [file 41467_2023_39425_MOESM3_ESM.pdf]

Description of Additional Supplementary Files

Supplementary Data 1:
TROSY-NMR spectra for pMLL/HLA-B*0702 and MLL/HLA-B*0702 complexes

Supplementary Data 2:
Binding between pMHC (pMLL/HLA-B*0702 complex) and TCR27, TROSY-NMR

Supplementary Data 3:
The top TCR-MHC docking solution coordinate file
