# Supplementary material for: Molecular mechanism of phosphopeptide neoantigen immunogenicity
Source: Nat Commun. 2023 Jun 23;14:3763. doi: 10.1038/s41467-023-39425-1 (PMC10290117; doi:10.1038/s41467-023-39425-1)
Supplement: Supplementary file 7 — Reporting Summary [file 41467_2023_39425_MOESM7_ESM.pdf]

nature portfolio

Corresponding author(s): Michelle Krogsgaard

Last updated by author(s): Mar 20, 2023

Reporting Summary

Nature Portfolio wishes to improve the reproducibility of the work that we publish. This form provides structure for consistency and transparency in reporting. For further information on Nature Portfolio policies, see our Editorial Policies and the Editorial Policy Checklist.

Statistics

For all statistical analyses, confirm that the following items are present in the figure legend, table legend, main text, or Methods section.

n/a	Confirmed	
☐	☒	The exact sample size (*n*) for each experimental group/condition, given as a discrete number and unit of measurement
☒	☐	A statement on whether measurements were taken from distinct samples or whether the same sample was measured repeatedly
☐	☒	The statistical test(s) used AND whether they are one- or two-sided<br>*Only common tests should be described solely by name; describe more complex techniques in the Methods section.*
☒	☐	A description of all covariates tested
☒	☐	A description of any assumptions or corrections, such as tests of normality and adjustment for multiple comparisons
☐	☒	A full description of the statistical parameters including central tendency (e.g. means) or other basic estimates (e.g. regression coefficient) AND variation (e.g. standard deviation) or associated estimates of uncertainty (e.g. confidence intervals)
☐	☒	For null hypothesis testing, the test statistic (e.g. *F*, *t*, *r*) with confidence intervals, effect sizes, degrees of freedom and *P* value noted<br>*Give P values as exact values whenever suitable.*
☒	☐	For Bayesian analysis, information on the choice of priors and Markov chain Monte Carlo settings
☒	☐	For hierarchical and complex designs, identification of the appropriate level for tests and full reporting of outcomes
☒	☐	Estimates of effect sizes (e.g. Cohen's *d*, Pearson's *r*), indicating how they were calculated

Our web collection on statistics for biologists contains articles on many of the points above.

Software and code

Policy information about availability of computer code

Data collection	X-ray data collection: SBCCollect https://sbc.aps.anl.gov/facilites<br>LSDC software, https://www.bnl.gov/nsls2/beamlines/beamline.php?r=17-ID-1<br>Flow cytometry - BD FACS Canto II System software v2.0, BD Fortessa LSR II DIVA v7.0
Data analysis	X-ray data processing, structure solution and refinement were from the CCP4 package v.8.0, and COOT 0.9.7<br>CCP4: software for Macromelcular X-Ray Crystallography, https://www.ccp4.ac.uk/<br>NMR data were processed using NMRPipe v.10.9 Rev 2021.139.10.46 (https://www.ibbrr.umd.edu/nmrpipe/install.html) and analyzed using NMRViewJ 9.2.0-b27(https://nmrfx.org)<br>Flow cytometry data were processed with FlowJo X and V10, Octet 9.1 was used to process bi-layer interferometry data and PYMOL 2.4 for Molecular visualisation.

For manuscripts utilizing custom algorithms or software that are central to the research but not yet described in published literature, software must be made available to editors and reviewers. We strongly encourage code deposition in a community repository (e.g. GitHub). See the Nature Portfolio guidelines for submitting code & software for further information.

Data

Policy information about availability of data

All manuscripts must include a data availability statement. This statement should provide the following information, where applicable:

- Accession codes, unique identifiers, or web links for publicly available datasets
- A description of any restrictions on data availability
- For clinical datasets or third party data, please ensure that the statement adheres to our policy

3D coordinates, structure factors and other relevant information about the crystal structures is available from the PDD under accession codes: 7S8J [https://www.rcsb.org/structure/7S8J] 7S8I [https://www.rcsb.org/structure/7S8I] 7S7E [https://www.rcsb.org/structure/7S7E] 7S7F [https://www.rcsb.org/structure/7S7F] 7RZD [https://www.rcsb.org/structure/7RZD] 7RZJ [https://www.rcsb.org/structure/7RZJ] 7S79 [https://www.rcsb.org/structure/7S79] 7S7D [https://www.rcsb.org/structure/7S7D] 7S8A [https://www.rcsb.org/structure/7S8A] 7S8E [https://www.rcsb.org/structure/7S8E] 7S8F [https://www.rcsb.org/structure/7S8F] NMR assignment data are available in the Biological Magnetic Resonance Data Bank, BMRB at https://bmrb.io/data_library/summary/index.php?bmrbId=51815 (deposition ID 51815). The sequences for TCR27 were deposited to Genebank, IDs MZ701715 and MZ701716.
Other data supporting the findings of this study are available within the article and its supplementary materials. Source data are provided as a Source Data file.

Research involving human participants, their data, or biological material

Policy information about studies with human participants or human data. See also policy information about sex, gender (identity/presentation), and sexual orientation and race, ethnicity and racism.

Reporting on sex and gender	N/A
Reporting on race, ethnicity, or other socially relevant groupings	N/A
Population characteristics	N/A
Recruitment	N/A
Ethics oversight	N/A

Note that full information on the approval of the study protocol must also be provided in the manuscript.

Field-specific reporting

Please select the one below that is the best fit for your research. If you are not sure, read the appropriate sections before making your selection.

☒ Life sciences ☐ Behavioural & social sciences ☐ Ecological, evolutionary & environmental sciences

For a reference copy of the document with all sections, see nature.com/documents/nr-reporting-summary-flat.pdf

Life sciences study design

All studies must disclose on these points even when the disclosure is negative.

Sample size	due to high reproducibility of the in vitro data (eg., T cell activation assays), the sample size =3 allows for adequate analysis and to reach a meaningful conclusion.
Data exclusions	No data were excluded.
Replication	All replications were successful, all measurements were replicated at least twice
Randomization	N/A
Blinding	Not relevant for in vitro experiments

Reporting for specific materials, systems and methods

We require information from authors about some types of materials, experimental systems and methods used in many studies. Here, indicate whether each material, system or method listed is relevant to your study. If you are not sure if a list item applies to your research, read the appropriate section before selecting a response.

Materials & experimental systems

n/a	Involved in the study
☐	☒ Antibodies
☐	☒ Eukaryotic cell lines
☒	☐ Palaeontology and archaeology
☒	☐ Animals and other organisms
☒	☐ Clinical data
☒	☐ Dual use research of concern
☒	☐ Plants

Methods

n/a	Involved in the study
☒	☐ ChIP-seq
☐	☒ Flow cytometry
☒	☐ MRI-based neuroimaging

Antibodies

Antibodies used	Anti-human CD3-FITC Clone UCTH1 BioLegend 300440<br>Anti-human CD4-PerCP/Cy5.5 Clone OKT4 BioLegend 317428<br>Anti-human CD8-PE Clone-RPA-T8 Biolegend 301051<br>Anti-human CD25-PE/Cy7 Clone BC96 BioLegend 302612<br>Anti-human CD69-APC Clone FN50 BioLegend 310910<br>Anti-human IFNγ-PE clone B27 Biolgend 506506
Validation	anti-human CD3: Verified Reactivity Human - Application / FC - Quality tested - - https://www.biolegend.com/en-gb/clone-search/fitc-anti-human-cd3-antibody-863<br>anti-human CD4 Antibody: Verified Reactivity Human, Cynomolgus, Rhesus - Application FC - Quality tested - https://www.biolegend.com/en-gb/products/percp-cyanine5-5-anti-human-cd4-antibody-5011<br>anti-human CD8a Antibody: Verified ReactivityHuman, Cynomolgus, Rhesus - Application FC - Quality tested - https://www.biolegend.com/en-gb/products/pe-anti-human-cd8a-antibody-836<br>anti-human CD25 Antibody: Verified ReactivityHuman - Application FC - Quality tested - https://www.biolegend.com/en-gb/products/pe-cyanine7-anti-human-cd25-antibody-1909<br>anti-human CD69 Antibody: Verified Reactivity Human - Application FC - Quality tested - https://www.biolegend.com/en-gb/products/apc-anti-human-cd69-antibody-1674<br>Anti-human IFNγ: Verified reactivity Human - Application / FC - Quality tested - https://www.biolegend.com/en-gb/clone-search/pe-anti-human-ifn-gamma-antibody-153

Eukaryotic cell lines

Policy information about cell lines and Sex and Gender in Research

Cell line source(s)	Tap-deficient T2 (174 x CEM.T2) cells (ATCC CRL-1991). Tap-deficient T2 (174 x CEM.T2) HLA-B7+ cells overexpressing HLA-B*07:02 were received from Dr. Engelhard, University of Virginia. Lymphoma-derived cell line that expresses low amounts of MHCI on the cell surface due to TAP deficiency and can only present exogenous peptides
Authentication	All cell lines were received from the reputable sources and used as is.
Mycoplasma contamination	All cell lines tested via IDEXX impact factor 3 or human and mouse pathogen
Commonly misidentified lines<br>(See ICLAC register)	No misidentified cell lines were used in the study

Flow Cytometry

Plots

Confirm that:

☒ The axis labels state the marker and fluorochrome used (e.g. CD4-FITC).

☒ The axis scales are clearly visible. Include numbers along axes only for bottom left plot of group (a 'group' is an analysis of identical markers).

☒ All plots are contour plots with outliers or pseudocolor plots.

☒ A numerical value for number of cells or percentage (with statistics) is provided.

Methodology

Sample preparation	Cells were collected to have a single cell suspension. Cells were washed, stained for dead cells, washed again. FC block was used to avoid aspecific staining. Cells were stained with antibodies at the dilution indicated by the manufacturer. Cells were

	obtained from cell culture plates.
Instrument	BD Fortessa LSR analyser BD Biosciences 647177
Software	BD FACSDive" and Flowjo X
Cell population abundance	T cells were 100% T cells as expanded population with anti-CD3/CD28. T2 cells were a cell line in culture so 100% pure.
Gating strategy	The global population of cells were gated on FSC-A and SSC-A. Then live T cells were gated from on a CD3A/Live. The live T cells population correspond to the negative population, CD3+. Singlets were then identified on a FSC-A/FSC-H plot. CD25+/CD69+ population were identified as positive for both markers on a same flow plot. The negative population used for control is a non-stimulated T cell population.

☒ Tick this box to confirm that a figure exemplifying the gating strategy is provided in the Supplementary Information.
